# Pneumoconiosis from Agricultural Dust Exposure among Young California Farmworkers

**DOI:** 10.1289/ehp.0800144

**Published:** 2009-02-25

**Authors:** Marc B. Schenker, Kent E. Pinkerton, Diane Mitchell, Val Vallyathan, Brenda Elvine-Kreis, Francis H.Y. Green

**Affiliations:** 1 Department of Public Health Sciences and; 2 Center for Health and the Environment University of California at Davis, Davis, California, USA; 3 Pathology and Physiology Research Branch, National Institute for Occupational Safety and Health, Morgantown, West Virginia, USA; 4 Department of Pathology and Laboratory Medicine, University of Calgary, Calgary, Alberta, Canada

**Keywords:** agriculture, dust, farmworker, interstitial fibrosis, pneumoconiosis, respiratory, small airways disease

## Abstract

**Background:**

Agricultural workers are exposed to airborne pollutants, including organic and inorganic (mineral) dusts.

**Objectives:**

Lung autopsy specimens from consecutive coroner’s cases of Hispanic males in Fresno County, California, (*n* = 112) were obtained to determine whether mineral dust exposure in agricultural work leads to pneumoconiosis.

**Methods:**

The left lung was fixed by inflation. We evaluated airway and parenchymal pathology using standardized diagnostic criteria and semiquantitative grading schemata, including the grading of small airways for fibrosis and birefringent mineral dust particles. We analyzed lung dust burden on a subset of 37 lungs following bleach digestion, using scanning electron microscopy (SEM), X-ray spectrometry (XRS) and image analysis, and by X-ray diffraction for crystalline silica (CSi). Farmworkers comprised 51.5% and nonfarmworkers 48.5% of the samples.

**Results:**

Proximal airways demonstrated little mineral dust accumulation, but membranous and respiratory bronchioles had wall thickening, remodeling, and inflammation associated with carbonaceous and mineral dust deposition. These changes were independently associated with agricultural work, cigarette smoking, and increased age. Mineral dust small airways disease, pneumoconiosis (macules and nodules), and pathologic changes consistent with chronic bronchitis, emphysema, and interstitial fibrosis predominated in farmworkers compared with nonfarmworkers. CSi, determined gravimetrically, and aluminum silicate particles, determined by SEM/XRS, were increased in the lungs of farmworkers compared with nonfarmworkers and were significantly (*p* < 0.05) associated with small airway disease and pneumoconiosis.

**Conclusion:**

Mineral dust exposure is associated with increased small airway disease and pneumoconiosis among California farmworkers; however, the clinical significance and natural history of these changes remains to be determined.

Approximately 5 million workers in the United States work in the agricultural industry with potential exposure to a wide variety of respiratory toxicants. Among the various respiratory hazards not well studied is obstructive and restrictive lung disease caused by inorganic minerals (American Thoracic Society 1998). California agricultural workers have increased respiratory symptoms, decreased respiratory function, and increased mortality rates from chronic pulmonary disease compared with the general population (Schenker et al. 2005; Singleton and Beaumont 1989; Stubbs et al. 1984).

The agricultural environment of the Central Valley of California places individuals at increased risk of exposure to inorganic particles. This region encompasses a rich farming area as well as extensive urban development. The predominantly dry farming techniques of the Central Valley result in high levels of airborne dust from operations such as field preparation and harvesting of row crops and tree fruits (Nieuwenhuijsen and Schenker 1998; Nieuwenhuijsen et al. 1998). Although soil consists of a mixture of organic and inorganic materials, potential health effects from the ever-present mineral dusts have been largely overlooked. In theory, soil should contain representative portions of all major mineral classes in the earth’s crust; in reality, most agricultural soils are composed largely of silicate materials (e.g., feldspars, mica, clay minerals) and crystalline silica (CSi) (quartz) with varying amounts of other mineral classes, depending on the local geologic history. The evidence that mineral dust exposure poses a significant hazard to agricultural workers for interstitial lung disease is based on a handful of case reports, inferences from exposures to mineral dusts in other industries, studies of wild and farm animals exposed to environmental dusts, and toxicologic studies (Gylseth et al. 1984; Schenker 2000; Schoning 1996; Vallyathan et al. 2007).

The deposition and clearance of particles within the respiratory system occurs in an inhomogeneous manner. The fate of particles is not well established, and there is little information on the distribution and retention of particles under conditions of ambient exposure. One purpose of this study was to design and implement an approach that would allow the assessment of particle retention as well as histologic analysis of response in different lung compartments. Our findings in this regard have been previously published and show that the deposition of particles is primarily in the centriacinar portion of the lung lobule (Pinkerton et al. 2000).

Unfortunately, no reported epidemiologic studies have directly assessed environmental exposure to mineral dusts and interstitial lung and airway disease in farmers or farmworkers, although respiratory symptoms have been associated with exposure to agricultural dusts with high mineral content (Schenker et al. 2005). A few studies, however, have suggested that pneumoconiosis (Gylseth et al. 1984; Sherwin et al. 1979) and restrictive lung disease (Sasagawa et al. 1989) are increased in some agricultural populations exposed to mineral dusts. A fundamental question remains whether occupational exposure to agricultural dusts can cause pulmonary fibrosis, persistent inflammation, and cell/tissue remodeling in specific regions of the lungs where these mineral dusts are present.

The objective of this study was to document and quantify pathologic lesions in lung tissues from consecutive Hispanic males autopsied by the coroner’s office in Fresno, California, and to determine their relationship to agricultural work and to mineral dust retained in the lungs. We focused on pneumoconiosis and lesions of the small airways. The latter are the primary target of mineral dusts (Churg et al. 1999; Craighead et al. 1982) and are most likely to reflect early disease in a young population (Pinkerton et al. 2000). We report that young male agricultural workers have a higher prevalence of pneumoconiosis and small airway disease associated with mineral dust exposure than do nonagricultural workers living in the same environment.

## Methods

### Population

Left lungs from 112 Hispanic male autopsies were collected at the Fresno County coroner’s office from June 1994 to June 1995. Demographic information including age, residence duration in Fresno County, and occupational histories were obtained from the medical examiner and the coroner’s files. Smoking histories were available for a minority of subjects; therefore, smoking status was classified by pathologic criteria. The study subjects ranged in age from 16 to 73 years and had died suddenly or unexpectedly. An autopsy was performed at the coroner’s office to determine the cause and manner of death, as dictated by state statute. The autopsies were performed within 12–24 hr after death. The research team independently evaluated a sagittal slice of lung. The project was thoroughly reviewed and approved by the Human Subjects Review Committee of the University of California, Davis. We did not have access to nor did we contact the next of kin.

### Tissue preparation

The left lung of each deceased individual was cannulated through the left mainstem bronchus and inflation-fixed with 2% glutaraldehyde at a hydrostatic pressure of 30 cm of water for 2 hr from a constant-pressure gravity apparatus. The lungs were cut in the sagittal plane to include the mainstem bronchus, hilar structures, and the medial aspect of both the upper and lower lobes of the left lung. If the left lung was not suitable because of trauma, the right lung was processed in an equivalent manner. Initial fixation and cutting was done by the coroner’s staff. The lung section was stored in fixative and shipped to the University of California, Davis. On arrival, each lung was photographed from the cut sagittal surface as well as a medial view, and selected gross features were documented on a standard form; these included pleural pigmentation, fibrosis, and emphysema. Selected airways were microdissected (Pinkerton et al. 2000) and examined for mucous plugs or aspirated material within the lumen.

### Histopathologic evaluation

**Figure 1:**
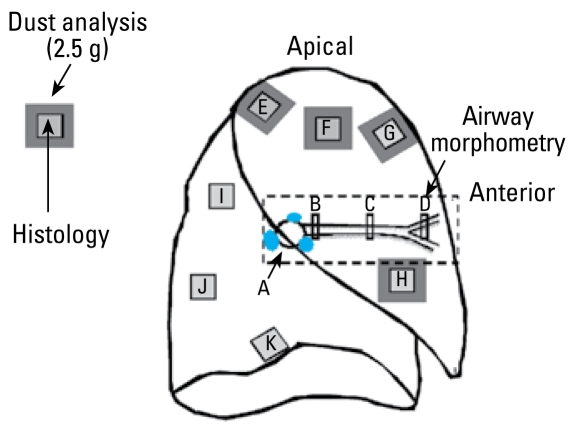
Sampling strategy for histopathologic and analytical lung tissue including conducting airways of varying sizes, central and peripheral lung parenchyma, and hilar lymph nodes. Histology was taken from sites A–K, samples for dust analysis from sites E, F, G, and H.

Samples of airways of varying size and airway generation, together with samples of pulmonary parenchyma, were taken from the upper and lower lobes using a standardized schema (Figure 1). Each tissue block was embedded in paraffin (Fischer Scientific, Fair Lawn, NJ, USA), and 5-μm-thick sections were cut using a rotary microtome and stained with hematoxylin and eosin. Sirius red and elastic trichrome stains were used to confirm the presence of collagen and smooth muscle within tissue sections.

We applied standard diagnostic criteria for the recognition of pneumoconiosis (Craighead et al. 1982, 1988) including macules, defined as collections of dust-laden macrophages within the walls of respiratory bronchioles but without significant fibrosis; nodules, defined as fibrotic lesions up to 1 cm in size with round, irregular, or serpiginous borders; and interstitial fibrosis.

The tracheobronchial lymph nodes were graded for fibrosis and dust. We evaluated pathologic changes consistent with chronic bronchitis and asthma using standard pathologic criteria (Green and Churg 1998a, 1998b). Small airways disease—subdivided into mineral dust-associated small airways disease (Churg 1992) and smoking-related small airways disease (Adesina et al. 1991; Cosio et al. 1980)—was diagnosed by accepted criteria. We assessed exposure to cigarette smoke in the recent past on the basis of accumulation of characteristic smokers’ macrophages within the respiratory bronchioles and adjacent alveoli (Niewoehner et al. 1974; Wright et al. 1992). Details of the diagnostic criteria for the lesions and the grading systems are provided in more detail in the Supplemental Material (available online at http://www.ehponline.org/members/2009/0800144/suppl.pdf).

### Mineral dust analysis

We used tissue samples from areas adjacent to those sampled for histology for the minerologic assays (Figure 1). The tissues were digested in filtered sodium hypochlorite, and the residual dust was analyzed by two methods. CSi was assayed by X-ray diffraction (XRD) by DataChem Laboratories (Salt Lake City, UT, USA) using NIOSH (National Institute for Occupational Safety and Health) method 7500. We analyzed separate aliquots by scanning electron microscopy (SEM) combined with X-ray spectrometry (XRS) and image analysis (Stettler et al. 1991) to determine elemental composition and particle size. For the SEM/XRS analyses, 1,000 or more inorganic particles were analyzed for each lung sample. Particles were placed into classes of minerals based on their elemental composition. Particles with > 90% silicon were classified as silica; particles with aluminum with or without other cations, that did not fit the criteria for silica, were classified as aluminum silicates (AlSi) (Stettler et al. 1991). Greater detail of the minerologic methods are given in the Supplemental Material (available online at http://www.ehponline.org/members/2009/0800144/suppl.pdf).

### Structural remodeling of small airways

The following airways were evaluated: membranous bronchioles and first-generation respiratory bronchioles cut in cross-sectional and perpendicular planes to the longitudinal profile of the airway, respectively. For respiratory bronchioles fibrosis, muscle hypertrophy, inflammation, intraluminal macrophages, opaque and birefringent particles were recorded. For membranous bronchioles, mucous cell hyperplasia was also recorded. Each feature was graded from 0 to 3, where 0 represented no evidence of that feature and 1 to 3 represented increasing grades of severity (Churg and Wright 1983). Variability of the readings and inter-observer reproducibility were established by having three readers (F.H.Y.G., K.E.P., and V.V.) each independently evaluate the same 30 histologic slides on two different occasions. More detail of the grading systems is given in the online Supplemental Material (available at http://www.ehponline.org/members/2009/0800144/suppl.pdf).

### Statistical analysis

We analyzed 112 male Hispanic cases for this study. Nine subjects were of undefined occupational status and were not included in comparisons of farmworker to nonagricultural occupation. Assessment of smoking was defined by histologic evidence of ever having smoked. Diseases that met the required pathologic criteria for their diagnosis [see Supplemental Material (available at http://www.ehponline.org/members/2009/0800144/suppl.pdf)] were coded dichotomously. Analysis used standard statistical methods and the SAS software procedures (SAS Institute Inc., Cary, NC, USA). Continuous variables are described by mean ± SD, median, quartiles, or ranges. Categorical variables are described using frequencies and percentages. Group comparisons for continuous variables were achieved using *t*-tests or analysis of variance for normally distributed data or the Kruskal–Wallis test for non-normally distributed data. For categorical data, we used chi-square analysis or logistic regression analysis to compare groups. We conducted bivariate analyses between independent variables and the outcome of interest. If the association between them generated a *p*-value < 0.05, the variable was considered for multivariate analysis.

We constructed logistic regression models to assess the association between disease states and variables of interest. Multivariate models always contained age and ever/never smoking status. Odds ratios (ORs) are given with 95% confidence intervals (CIs). We used linear regression models to assess the association between continuous independent variables, such as the abundance of carbonaceous substances or crystalline dust, and dependent variables. We used general linear models to determine the association between worker classification and the amount of mineral deposition in lung tissue (least mean squares), with age adjustment. Interaction terms were included in multivariate regression models if the interaction term was significant at the *p* ≤ 0.05 level and confounders if the OR or the point estimate was altered by ≥ 15%.

## Results

### Study population

All cases were Hispanic males with a mean age of 32.5 years (range, 16–73 years) (Table 1). Approximately one-third had lived in Fresno County ≤ 10 years, one-third for 11–20 years, and one-third for ≥ 20 years. Overall education was low (mean = 8.1 years), and agricultural workers had significantly less education than nonagricultural workers. Approximately half of the subjects were classified as current smokers at the time of death (Table 1). Cause of death was classified according to the *International Classification of Diseases, 9th Revision, Clinical Modification* (ICD-9CM; U.S. Department of Health and Human Services 2002) (Table 2). The predominant causes of death were vehicular accidents (50%), homicide (21%), cardiovascular disease (10%), and suicide (8%).

### Lung pathology

Gross examination revealed varying amounts of black pigmentation in the pleura, around bronchovascular bundles, in the centriacinar zones of the parenchyma, and within hilar lymph nodes. Airway microdissection showed that dust accumulation was less proximally but became distinct around small airways. Grossly recognizable emphysema was rarely seen. Many lungs showed parenchymal hemorrhage consistent with a traumatic death.

Smoking-related small airway disease and mineral dust–associated small airways disease were seen in 54.5% and 28.6% of all cases, respectively (Table 3). Pneumoconiosis (macules and/or nodules) was observed in 20.9% of subjects, lymph node fibrosis associated with mineral dust accumulation in 48.7%, pathologic changes consistent with chronic bronchitis in 56.3%, and microscopic emphysema in 23.6%. Asthmalike inflammation and airway wall remodeling were seen in 26.8% of 112 subjects (Table 3). The crude prevalence of mineral dust small airways disease, pneumoconiosis, and pathologic changes consistent with chronic bronchitis was significantly (*p* < 0.05) higher among farmworkers than among nonagricultural workers and approached statistical significance for lymph node fibrosis and emphysema.

In univariate models of the relationship between pathologic disease and mineral dust deposition as evaluated by polarized light microscopy on tissue sections, mineral dust deposition was strongly and significantly associated with interstitial fibrosis, mineral dust small airway disease, pneumoconiosis, pathologic changes consistent with chronic bronchitis, emphysema, and lymph node fibrosis (Table 4). These associations remained significant after adjustment for age and smoking status. Cigarette smoking was associated with an OR of < 1 for mineral dust small airways disease, but this association was small compared with the very strong association with mineral dust exposure (OR = 575.4; 95% CI, 39.4 to > 999). Agricultural work was kept in the model for chronic bronchitis over mineral dust because it had a higher point estimate (OR = 2.58; 95% CI, 0.87–7.72), although it did not achieve statistical significance at *p* < 0.05.

**Figure 2:**
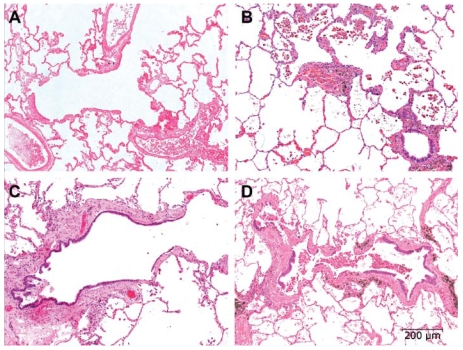
Composite photomicrographs of representative examples of first-generation respiratory bronchioles. All images were taken at the same magnification; bar = 200 μm. (*A*) Proximal respiratory bronchiole from a 43-year-old nonsmoking, nonagricultural worker. There is no evidence of fibrosis or inflammation. The airway has divided into three separate segments, each attended by a small pulmonary artery. Grade 0 for all features. (*B*) Proximal respiratory bronchiole from an 18-year-old nonagricultural worker who smoked. The respiratory bronchiole and its bifurcations shows thickening of the wall by fibrosis, muscular hypertrophy, chronic inflammation, and numerous smoker’s macrophages within the lumen of the airway and adjacent alveoli. This airway was considered grade 2 for fibrosis. (*C*) Proximal respiratory bronchiole from a 56-year-old nonsmoking agricultural worker. The airway wall shows marked thickening, mild inflammation, and no smoker’s macrophages in the alveoli. This bronchiole was considered grade 3 for fibrosis. (*D*) Proximal respiratory bronchiole from a 23-year-old agricultural worker who smoked. The airway wall shows marked thickening, moderate to severe inflammation, and numerous smoker’s macrophages in the lumen of the airway and adjacent alveoli. This bronchiole was considered grade 3 for fibrosis.

**Figure 3:**
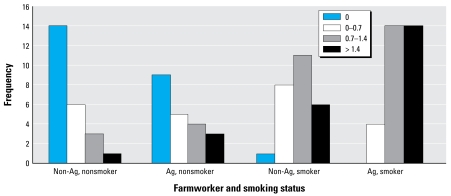
Fibrosis score (by quartiles) of agricultural work and smoking status for first-generation respiratory bronchioles. Abbreviations: Ag, agricultural; Non-Ag, nonagricultural. Increasing scores for fibrosis were associated with both agricultural work and smoking status. The effects of cigarette smoke and farm dust exposure appear additive.

Fibrosis of the walls of membranous and respiratory bronchioles was seen in most of the subjects. Examples of airway lesions in the groups are shown in Figure 2. The fibrosis was significantly (*p* < 0.05) greater in the upper lobes compared with the lower lobes. Forty-one percent of the nonsmoking, nonagricultural workers showed no fibrosis of their respiratory bronchioles (frequency score of 0) (Figure 3). Very few nonagricultural workers exhibited a severe grade of airway fibrosis (frequency score > 1.4). Agricultural workers had more severe grades of fibrosis. The severity of the small airway disease (respiratory bronchioles and membranous bronchioles) increased in the following order: nonsmoking nonagricultural workers; nonsmoking agricultural workers; smoking nonagricultural workers; smoking agricultural workers. The effects of smoking and agricultural work on grade of small airway disease appeared additive (Figure 3). By bright field and polarized light microscopy, opaque and birefringent dust in farmers’ respiratory bronchioles was highly correlated with small airways fibrosis (*r* = 0.66).

There was a highly significant relationship between agricultural work and a finding of pneumoconiosis (Table 5): A total of 32.1% of agricultural workers had either macules or nodules in their lungs compared with only 8.3% of nonagricultural workers (*p* = 0.003). is association persisted in a multivariate analysis controlling for age and cigarette smoking. Prevalence of pneumoconiosis increased to 41.5% and 18.6% in agricultural and nonagricultural workers, respectively, when interstitial fibrosis was included as a feature of pneumoconiosis (*p* < 0.0001). Pneumoconiosis was also significantly associated with an increased score for birefringent mineral particles in the walls of small airways (Table 5). The correlation between pneumoconiosis and the mineral dust score was *r* = 0.57. In multivariate models that controlled for age and cigarette smoking, agricultural work was a significant independent predictor of pneumoconiosis (OR = 5.362; 95% CI, 1.547–18.58) (Table 6).

CSi and AlSi were the most prevalent exogenous minerals found in the lungs for all groups (Table 7). Milligrams of quartz per 100 grams of lung, measured by XRD analysis, were significantly (*p* < 0.05) increased in agricultural workers who smoked compared with nonsmoking nonagricultural workers and in nonagricultural smokers compared with nonagricultural nonsmokers (Table 7). Logistic regression analysis, adjusted for age and smoking, showed a significant relationship between the amount of quartz determined gravimetrically in the lung and the presence of mineral dust small airway disease (OR = 1.114; 95% CI, 1.007–1.233) (*p* < 0.05). SEM/XRS analysis showed greater numbers of total mineral particles, silica particles, and AlSi workers compared with nonagricultural workers and in smokers compared with nonsmokers (Table 7). The sizes of the mineral dust particles by type of particle and subject group are also shown in Table 7. The average median circular equivalent diameters for silica and silicate particles were < 1 μm for all four groups. Silicate particles were significantly (*p* < 0.05) larger than silica particles overall, with the greatest difference seen in the nonsmoking agricultural workers (Table 7). Mineral dust small airway disease was significantly (*p* < 0.05) associated with the number of silica, AlSi, and total mineral dust particles in the lung in univariate models and with total particle number in multivariate models.

## Discussion

The lungs of deceased farmworkers living in an agricultural region of California had significantly higher rates of pneumoconiosis and interstitial fibrosis than the lungs of deceased nonagricultural workers living in the same general environment. Furthermore, the farmworkers showed higher prevalence of chronic obstructive pulmonary disease (COPD), including emphysema, pathologic changes consistent with chronic bronchitis, and small airways disease than the lungs of deceased nonagricultural workers living in the same general environment. Multivariate analyses showed that these pathologic lesions were strongly associated with mineral dust in the lungs, as assessed by grade of birefringent particles in the small airways, the amount (milligrams per gram) of quartz, and number of silica and silicate mineral dust particles in digested lung samples. The study indicates that agricultural work in the central valley of California carries a significant risk for mineral dust small airway disease, pneumoconiosis, and COPD.

The relationship between mineral dust exposure, small airway disease, and pneumoconiosis was confirmed by three separate approaches. First, and most important, by light microscopy we directly confirmed a relationship between airway fibrosis, pneumoconiosis, and birefringent mineral particles. Second, in a subgroup of the cases, we showed, in bulk samples of lung tissue, that CSi was significantly associated with small airway fibrosis. Third, we showed by SEM and XRS that the total number of mineral particles (silica and silicates) was greater in the lungs of agricultural workers than in nonagricultural workers and that their concentration was significantly associated with airway fibrosis. We thus demonstrate that mineral dusts play an important role in lung disease for agricultural workers and that mineral dusts need to take their place beside the well-established roles of organic dusts and smoking in causing lung disease in farmworkers. Our data also indicate that mineral dusts contribute to both obstructive and restrictive lung disease processes.

The findings reported here are remarkable in view of the young age of the study population. Mineral dust pneumoconioses are generally considered to have long latencies until clinically apparent, on the order of 10–20 years (Smith and Leggat 2006). The latency period until early pathologic changes that are not radiologically or clinically evident is unknown. The cases examined in this study were young Hispanic males who had lived an average of 16 years in Fresno County. Approximately one-half of these subjects were farmworkers, whereas the others were in other blue-collar occupations. None of these individuals died of respiratory disease, and most were in apparent good health before death.

This is the first population-based sample that we are aware of that shows small airway and interstitial lung disease associated with agricultural work. Case–control studies have suggested an association of idiopathic pulmonary fibrosis (IPF) with agricultural dust exposure, and specifically with animal dust/vegetable dust, but these studies have involved multiple associations without verification of exposure (Baumgartner et al. 2000). A case–control study of inorganic particles in the pulmonary hilar lymph nodes of patients with IPF found an association of increased silicon and aluminum compared with control lymph nodes (Kitamura et al. 2007). A study using SEM and energy-dispersive X-ray analysis suggested that silica/silicate exposure might be a risk factor for IPF (Monso et al. 1990). IPF is also strongly associated with cigarette smoking in epidemiologic studies (Taskar and Coultas 2006). Similar to our findings, Nasr et al. (2006) have recently shown that cigarette smokers with interstitial fibrosis have increased burdens of silica and silicates in their lungs. Taken together, these studies indicate that exposure to inorganic dusts, whether overt or occult, may be a more common cause of pulmonary fibrosis than currently recognized and that the term “idiopathic” may be an inaccurate description for some cases of IPF.

The prevalence of histologic-determined cigarette smoking in this population was 54.5%, which is significantly higher than population-based data on smoking prevalence among Hispanic males (Perez-Stable 2001). However, smokers have excess injury deaths (RR = 1.86), which are heavily weighted in coroner’s cases (Leistikow et al. 2000; Wen 2005). Furthermore, young Hispanics living in the United States have higher rates of smoking than non-Hispanic youth (Perez-Stable 2001). There also may be some degree of misclassification of smoker status, as the diagnosis of recent exposure to cigarette smoke was based on the presence of clusters of characteristic smoker’s macrophages in the alveoli adjacent to the respiratory bronchioles. Although not well documented for humans, it is likely that high and prolonged exposure to diesel emissions or burning biomass could produce similar changes in the lung. These types of exposures are relatively common in the farming industry. Misclassification of smoking status would not affect the relationships we saw between agricultural work, mineral dust in the lung, mineral dust small airway disease, and pneumoconiosis.

The statistically significant association of smoking with reduced odds of mineral dust small airways disease (Table 4) is of unknown significance. Cigarette smoking was associated with increased odds for other respiratory health outcomes, as expected. Examination of Figure 3 shows that farm work and smoking have additive effects on the milder grades of small airway fibrosis. However the data also indicate that there is a maximal response (threshold effect) to these agents that might account for the negative interaction in the multivariate analyses.

Morbidity and mortality studies in several countries have observed an association of COPD and agricultural work (Brackbill et al. 1994; Lamprecht et al. 2007; Schenker 1998), but this is not observed in all agricultural populations (Blair et al. 2005). This association is observed concomitant with a lower mortality rate from lung cancer, reflecting the lower known smoking prevalence among agricultural workers (Singleton and Beaumont 1989). We observed a strong association of mineral dust exposure with interstitial fibrosis and pneumoconiosis, after adjustment for cigarette smoking. However, we also found independent associations of mineral dust exposure and emphysema, chronic bronchitis, lymph node fibrosis, and mineral dust small airways disease. Our findings suggest one possible mechanism for a causal association of agricultural work and COPD: exposure to elevated mineral dust levels.

We have previously documented very high occupational exposures to respirable particles among agricultural workers in California’s central valley (Nieuwenhuijsen et al. 1998, 1999). For example, personal dust exposure levels measured during various operations such as land planning and disking yielded geometric mean particle concentrations of 57.3 and 98.6 mg/m^3^, respectively. Although these samples were weighted toward large particles, respirable particle concentrations of 1–3 mg/m^3^ were nevertheless common.

Situated in the heart of the San Joaquin Valley, Fresno has some of the highest ambient inhalable particle concentrations (particulate matter ≤ 10 μm; PM_10_) in the United States, often exceeding the National Ambient Air Quality Standard of 150 μg/m^3^ averaged over 24 hr (U.S. Environmental Protection Agency 2006).

Chow et al. (1992, 1993) describe in detail the physicochemical characteristics and seasonal variability of PM in Fresno. During the winter months, the highest PM_10_ (particulate matter with aerodynamic diameter < 10 μm) concentrations have a dominant PM_2.5_ fraction (i.e., fraction of particles < 2.5 μm in aerodynamic diameter). These particles consist mostly of carbonaceous constituents (especially particles < 0.3 μm) as well as ammonium nitrate and sulfate (0.3–2.5 μm). Elevated PM_10_ concentrations during the summer and early fall occur because of windblown dust excursions, which have been found most often in the southern San Joaquin Valley and in the high desert regions. These situations are dominated by fugitive dusts, mostly associated with coarse (i.e., 2.5–10 μm) particles.

From 14 June 1994 through 9 June 1995, when the autopsy material was collected for this study, California and federal 24-hr and annual standards for PM_10_ were regularly exceeded in Fresno County at both urban and nonurban sites (Alexis et al. 1999). During this time, the PM_10_ daily (arithmetic) average concentration in Fresno was 43.5 μg/m^3^, and the maximum 24-hr PM_10_ concentration was 122 μg/m^3^. The corresponding levels of PM_2.5_ were 22 and 65 μg/m^3^. Oxidant gases were also measured during this period. One-hour measurements of nitrogen oxides (NO_x_) averaged 0.109 ppm, with maximal levels reaching 0.7 ppm. The average 1-hr concentration of ozone during this time was 0.06 ppm, whereas the maximum 1-hr ozone concentration was 0.17 ppm. Sulfur oxide levels averaged 0.0054 ppm with a maximal concentration of 0.017 ppm (Alexis et al. 1999).

In a previous study involving a subset of this population, we used a systematic approach to determine the distribution of ambient particles in the human lung and their potential role in tissue remodeling (Pinkerton et al. 2000). is approach involved dissection of defined airway paths and parenchymal sampling in adjacent regions. As a result of this sampling procedure, the importance of terminal and respiratory bronchioles as sites for particle retention and the association of particle retention with subtle but quantifiable changes in tissue remodeling were clearly established. The importance of this site as a target for particle-induced injury is well established in occupational settings (Green 1983). The centriacinar region is the primary site of injury in coal worker’s pneumoconiosis (Kleinerman et al. 1979), asbestosis-induced injury (Craighead et al. 1982), and silica- and silicate-induced injury (Craighead et al. 1988). Case reports of silicate pneumoconiosis have been reported in farmworkers from the central valley of California (Sherwin et al. 1979).

Cigarette smoking (Niewoehner et al. 1974) and highly polluted urban centers (Churg et al. 2003) also induce injury in this region, and there is considerable evidence that respiratory bronchiolitis precedes the functionally more important disease of centriacinar emphysema by several decades (Wright et al. 1992). However, it is not generally recognized that subtle lesions can occur in the centriacinar zone of the lung in individuals exposed to ambient particles.

Our results show a continuum of changes in the respiratory and membranous bronchioles, being most severe in individuals who smoke and in persons exposed to agricultural dusts, but also present to a lesser degree in the population in general. The latter is not surprising in view of the poor air quality in the region (Alexis et al. 1999). We do not have data on lifetime exposures, but both the agricultural workers and nonagricultural workers would likely have had similar exposure to ambient air pollutants. The high levels of ozone in this region may also be contributing to the background levels of airway disease (Harkema et al. 1993).

The physiologic effects of the small airway lesions are uncertain, because subtle changes such as those shown in this study can be detected only by nonroutine tests for small airway function (Wright et al. 1992). However, the changes described here in these young farmworkers may not be trivial, as they can progress to clinically significant disease (Wright et al. 1992).

## Conclusion

Agriculture dust exposure is associated with mineral dust small airway disease and pneumoconiosis, independent of age and cigarette smoking. These changes were observed in young individuals with no known respiratory disease. The natural history of this lesion in agricultural workers is unknown but deserves further study in longitudinal investigations of agricultural workers, using techniques sensitive to detection of small airway disease and pneumoconiosis.

## Figures and Tables

**Table 1:** Demographic characteristics of population.

Characteristic	Agricultural worker	Nonagricultural worker	Overall	Significance of difference between agricultural and nonagricultural workers^a^
Total no. of subjects (%)	53 (51.5)	50 (48.5)	103/112	(9 unknown status)
Ever smoked^b^	32 (60.4)	26 (52.0)	58 (56.3)	Not significant
				χ^2^ = 0.74
				*p* = 0.39
Age (years)	35 ± 14.2	30 ± 10.5	32.5 ± 12.8	χ^2^ = 3.18
				*p* = 0.075
Years education	5.6 ± 3.2	10.1 ± 2.8	8.1 ± 3.7	χ^2^ = 35.8
				*p* ≤ 0.0001
Years in Fresno County	11.9 ± 11.0	17.2 ± 12.0	14.95 ± 11.9	χ^2^ = 5.98
				*p* = 0.0145

Values are no. (%) or mean ± SD unless noted otherwise. All subjects were male and Hispanic (total *n* = 112).

a Chi-square categorical tests used except for age and years of education, where the Kruskal–Wallis test giving a chi-square value was used.

b Based on histologic criteria (see text).

**Table 2:** Cause of death (total *n* = 112).

Cause of death	ICD-9CM code range	Frequency (%)^a^
Heart and cardiovascular disease	410–429	12 (10)
All vehicle accidents	E800–E848	54 (50)
Accidental poisonings by analgesics/psychotropic agents	E850–E854	5 (4)
Excessive heat	E900	1 (1)
Accidental drowning and submersion	E910	4 (4)
Agricultural machine accident	E919	1 (1)
Accidental electrocution	E925	1 (1)
Suicide and self-inflicted injury	E950–E959	9 (8)
Homicide and injury inflicted by others	E960–E969	23 (21)
Unknown cause	—	2 (2)

a Percentages may not add to 100 because of rounding.

**Table 3:** Global diagnoses based on lung pathology in 112 residents of Fresno County, California, USA.

	Prevalence (%)			
Specific feature	All cases	Farmworkers	Non-farmworkers	*p-*Value
Mineral dust small airways disease	32 (28.6)	22 (41.5)	9 (18.0)	0.009
Smoking-related small airways disease	61 (54.5)	32 (60.4)	26 (52)	0.392
Pneumoconiosis (macules and/or nodules)	23 (20.9)	17 (32.1)	4 (8.3)	0.003
Interstitial fibrosis	21 (19.1)	13 (24.5)	7 (14.6)	0.210
Lymph node fibrosis	54 (48.7)	30 (56.6)	19 (38.8)	0.072
Chronic bronchitis^a^	63 (56.3)	36 (67.9)	24 (48.0)	0.040
Asthma-like airway disease^b^	30 (26.8)	12 (22.6)	14 (28.0)	0.530
Emphysema	26 (23.6)	17 (32.1)	8 (16.7)	0.073

a Defined on histologic criteria as pathologic changes consistent with chronic bronchitis.

b Defined on histologic criteria, including the presence of all of the following: smooth muscle hyperplasia/hypertrophy, goblet cell metaplasia, mucous gland enlargement, thickening of basement membrane, and chronic inflammation with lymphocytes and occasional eosinophils.

**Table 4:** Associations between disease, agricultural work, and mineral dust in small airways: logistic regression [OR (95% CI)].

	Univariate association OR (95% CI)		Multivariate association (Adjusted for age and smoking status)^a^	
Disease category	Agricultural work	Mineral dust deposition	Variable	OR (95% CI)
Interstitial fibrosis	1.90 (0.69 to 5.26)	12.35 (3.39 to 44.90)	Mineral dust	6.21 (1.39 to 27.62)
			Age	1.05 (1.01 to 1.10)
			Smoking status	5.03 (1.12 to 22.68)

Mineral dust small airways disease	3.23 (1.31 to 7.99)	161.8 (21.4 to > 999)	Mineral dust	575.4 (39.4 to > 999)
			Age	1.035 (0.99 to 1.08)
			Smoking status	0.116 (0.022 to 0.618)^b^

Pneumoconiosis	5.19 (1.6 to 16.82)	166.8 (17.56 to > 999)	Mineral dust	453 (24.96 to > 999)
			Age	0.96 (0.914 to 1.013)
			Smoking status	6.09 (0.88 to 41.904)

Chronic bronchitis^c^	2.29 (1.03 to 5.10)	4.68 (1.75 to 12.52)	Agricultural worker^d^	2.58 (0.87 to 7.72)
			Age	1.032 (0.99 to 1.08)
			Smoking status	23.93 (8.00 to 71.54)

Emphysema	2.36 (0.91 to 6.13)	16.08 (4.35 to 59.46)	Mineral dust	5.62 (1.04 to 30.24)
			Age	1.14 (1.07 to 1.25)
			Smoking status	9.63 (1.66 to 55.94)

Lymph node fibrosis	2.06 (0.93 to 4.54)	9.15 (3.11 to 26.9)	Mineral dust	12.54 (3.04 to 51.7)
			Age	1.03 (0.99 to 1.07)
			Smoking status	0.43 (0.15 to 1.20)

a Multivariate models always contained age and smoking status unless otherwise specified. They were checked for interactions between smoking status, grade of birefringent pigment in airway walls, and age. The model with both agricultural work and birefringent pigment was kept if both variables were significant or if one acted as a confounder to the other (changed the adjusted point estimate > 15%). Smoking status association was of similar size and significance whether the more inclusive variable ever smoked was used or current smoker, which excluded nine subjects.

b Smoking protective against mineral dust small airway disease.

c Defined by pathologic criteria.

d Agricultural worker was kept in the model over mineral dust as the point estimate was larger and CI tighter, but both were inferior to smoking status for odds of chronic bronchitis. The univariate association with smoking status was 21.01 (7.94–55.57).

**Table 5:** Associations of pneumoconiosis^a^ with agricultural work, smoking, and mineral dust particles in small airways: univariate analysis.

Exposure	Pneumoconiosis
Agricultural work (%)	32.1
No agricultural work	8.33
Chi-square, *p*-value	χ^2^ = 8.62, *p* = 0.0033
Never smoked (%)	8.16
Ever smoked	31.2
Chi-square, *p*-value	χ^2^ = 8.68, *p* = 0.0032
Mineral dust particles (%)	
Score < 0.25	0
0.25–0.9	8.62
0.9–2.03 (max)	66.7
Chi-square, *p*-value	χ^2^ = 46.1, *p* = < 0.0001

a Defined as presence of macules and/or nodules.

**Table 6:** Independent association of pneumoconiosis with agricultural work: multivariate logistic regression.

Dependent variable	Covariate	OR (95% CI)
Pneumoconiosis	Age	1.001 (0.962–1.042)
	Smoking	6.208 (1.612–23.91)
	Agricultural work	5.362 (1.547–18.58)

**Table 7:** Age-adjusted mineral content of the lung by agricultural work and cigarette smoking status (mean ± SE).

Mineral component	No.	[1] Nonagricultural nonsmoker	[2] Nonagricultural smoker	[3] Agricultural worker nonsmoker	[4] Agricultural worker smoker	Significant differences^a^
Mg quartz/100 g lung^b^	35	5.13 ± 3.82	18.87 ± 4.08	12.31 ± 3.42	18.02 ± 3.42	1:2, 1:4^c^
SEM/XRS analyses:						
Particles analyzed per case	37	1505.4 ± 178	1,238 ± 167	1,051 ± 149	1,457 ± 178	None
Total mineral particles per gram dry lung	37	20.83 ± 16.30	38.83 ± 16.30	33.94 ± 15.37	101.79 ± 13.31	4:1, 4:2, 4:3
No. of silica particles per gram dry lung	37	16.83 ± 6.92	31.78 ± 6.93	25.18 ± 6.53	45.33 ± 5.65	4:1, 4:3
Median circular equivalent diameter silica (μm)	36	0.52 ± 0.05	0.48 ± 0.05	0.51 ± 0.05	0.50 ± 0.04	None
No. of AlSi particles per gram dry lung	37	4.00 ± 12.86	7.05 ± 12.86	8.76 ± 12.12	56.46 ± 10.50	4:1, 4:2, 4:3
Median circular equivalent diameter (AlSi) (μm)	35	0.68 ± 0.08	0.50 ± 0.10	0.80 ± 0.08	0.57 ± 0.07	3:2, 3:4
Difference in size between silica and AlSi particles	36	0.16 ± 0.07	0.02 ± 0.08	0.29 ± 0.07	0.07 ± 0.06	3:2, 3:4

Two outliers were removed from these analyses, both nonagricultural workers. One of these had widespread granulomatous lung disease, the other had worked as a stonemason. Codes 1–4 for different categories are represented in the final column as significant differences.

a Differences between adjusted means *p* < 0.05.

b This association became nonsignificant when age was added into the model. All other associations remained significant.

c XRD.
